# EP1^−/−^ Mice Have Enhanced Osteoblast Differentiation and Accelerated Fracture Repair

**DOI:** 10.1002/jbmr.272

**Published:** 2010-10-11

**Authors:** Minjie Zhang, Hsin-chiu Ho, Tzong-jen Sheu, Matthew D Breyer, Lisa M Flick, Jennifer H Jonason, Hani A Awad, Edward M Schwarz, Regis J O'Keefe

**Affiliations:** 1 Center for Musculoskeletal Research, University of RochesterRochester, NY, USA; 2 Department of Biomedical Genetics, University of RochesterRochester, NY, USA; 3 Biotechnology Discovery Research, Eli Lilly and CompanyIndianapolis, IN, USA

**Keywords:** EP1, FRACTURE HEALING, CHONDROCYTE, CHONDROCYTE MATURATION, MINERALIZATION, OSTEOBLASTS, OSTEOCLASTS, PGE_2_

## Abstract

As a downstream product of cyclooxygenase 2 (COX-2), prostaglandin E_2_ (PGE_2_) plays a crucial role in the regulation of bone formation. It has four different receptor subtypes (EP1 through EP4), each of which exerts different effects in bone. EP2 and EP4 induce bone formation through the protein kinase A (PKA) pathway, whereas EP3 inhibits bone formation in vitro. However, the effect of EP1 receptor signaling during bone formation remains unclear. Closed, stabilized femoral fractures were created in mice with EP1 receptor loss of function at 10 weeks of age. Healing was evaluated by radiographic imaging, histology, gene expression studies, micro–computed tomographic (µCT), and biomechanical measures. EP1^−/−^ mouse fractures have increased formation of cartilage, increased fracture callus, and more rapid completion of endochondral ossification. The fractures heal faster and with earlier fracture callus mineralization with an altered expression of genes involved in bone repair and remodeling. Fractures in EP1^−/−^ mice also had an earlier appearance of tartrate-resistant acid phosphatase (TRAcP)–positive osteoclasts, accelerated bone remodeling, and an earlier return to normal bone morphometry. EP1^−/−^ mesenchymal progenitor cells isolated from bone marrow have higher osteoblast differentiation capacity and accelerated bone nodule formation and mineralization in vitro. Loss of the EP1 receptor did not affect EP2 or EP4 signaling, suggesting that EP1 and its downstream signaling targets directly regulate fracture healing. We show that unlike the PGE_2_ receptors EP2 and EP4, the EP1 receptor is a negative regulator that acts at multiple stages of the fracture healing process. Inhibition of EP1 signaling is a potential means to enhance fracture healing. © 2011 American Society for Bone and Mineral Research.

## Introduction

Approximately 7.9 million skeletal fractures occur each year in the United States.(1) Nearly 10% to 20% of all fractures have impaired healing, including delayed union or nonunion. Impaired fracture healing, which requires prolonged or repeated treatments, has a marked impact on both the quality of life and the total cost of care. Since the risk of impaired fracture healing is greatest in the elderly and those with other infirmities, the reduced mobility that occurs with fractures further complicates the management of these other conditions, resulting in additional personal and societal costs.(2) For this reason, identifying targets and potential therapies to enhance the rate of fracture healing has great importance.

Nonsteroidal anti-inflammatory drugs (NSAIDs) taken to reduce pain following fracture have been linked to decreased bone repair in several clinical studies.(3,4) NSAIDs inhibit the activity of cyclooxygenase (COX) enzymes COX-1 and COX-2. These enzymes play a critical role in the synthesis of prostaglandins (PGs) from arachidonic acid. While COX-1 is constitutively expressed and plays a largely homeostatic role in bone, COX-2 expression is induced by mitogens and inflammatory cytokines to upregulate PG synthesis during repair as well as in settings of inflammation and tumorigenesis.(5,6) Several experiments have confirmed that NSAID inhibition of cyclooxygenase 2 (COX-2) during the inflammatory stage of healing delays the rate of fracture repair.(7–9) Prior studies show significant delay in fracture healing in *COX-2* null mice compared with wild-type littermates and *COX-1* null mice.(10,11) *COX-2*^−/−^ mice have reduced callus formation, delayed chondrogenesis and impaired endochondral bone formation, and reduced vascularization, primary bone formation, and remodeling.(10) However, the role of COX-2/PGE_2_ in humans has remained controversial because other reports describe no reduction in the rate of fracture healing following administration of NSAIDs.(12–14)

As the major downstream product of COX-2, PGE_2_ plays a crucial role in regulating bone formation. PGE_2_ exerts its effects through binding to four different G protein–coupled 7-transmembrane receptors, and thus the overall effect of PGE_2_ on fracture healing is due to a combination of signaling events downstream of these receptors.(15) The EP2, EP3, and EP4 receptors each modulate cAMP levels.(16,17) EP2 and EP4 activation stimulates the production of cAMP through Gs. In contrast, EP3 activation results in a decrease in cAMP levels through Gi, Gq, or Gs, depending on the EP3 isoform. Less is known about the EP1 receptor. While the EP1 receptor is involved in regulating intracellular calcium levels, the G protein to which it couples remains to be identified.

EP2 and EP4 receptor knockout mice have been shown to have impaired fracture healing and impaired bone resorption.(18,19) An EP2-selective agonist induced bone healing in beagles(20); similarly, an EP4-selective agonist has been used in a rat model of bone repair with positive effects.(21,22) Recent work in our laboratory showed that another EP4-selective agonist accelerated the delayed fracture healing in aged mice and compensated for the reduced fracture healing observed in *COX-2*^−/−^ mice.(23,24) In contrast, treatment of fractures in *COX-2*^−/−^ mice with an EP2 agonist enhanced fracture repair only marginally.(23) Furthermore, while the EP4 agonist significantly increased cartilage nodule formation in E11.5 murine limb bud cultures similar to PGE_2_, the EP2 agonist had no effect on chondrogenesis. Thus the different EP receptors appear to mediate unique effects on the cells and tissues involved in fracture repair. Indeed, the EP3 receptor, which negatively regulates cAMP levels, is suggested to have negative effects on bone formation.(25)

Although EP1 receptor expression in bone was reported more than a decade ago, its role in bone metabolism is less clear. EP1 agonists were shown to stimulate proliferation and inhibit differentiation in MC3T3-E1 cells.(26) EP1 agonists also stimulate growth plate chondrocyte proliferation and influence osteoclast differentiation in murine bone marrow cell cultures.(27,28) EP1 agonist treatment in rat osteoblast cultures stimulates fibronectin expression.(29) While the work to date clearly demonstrates that EP1 modulates osteoblast gene expression and function, there are no studies defining the role of EP1 in fracture repair. While *EP1*^−/−^ mice are not dramatically different from wild-type mice in terms of skeletal strength or size,(18) a similar observation can be made for *COX-2*^−/−^ mice, which have marked impairment of fracture healing.(10,11,30)

In this study, we observed accelerated fracture healing in *EP1*^−/−^ mice compared with wild-type mice. Fractures in *EP1*^−/−^ mice have more rapid healing, accelerated fracture callus mineralization and bone remodeling, and earlier and increased expression of chondrocyte and osteoblast-specific genes. *EP1*^−/−^ bone marrow cell cultures undergo more rapid differentiation and mineralization and have enhanced expression of osteoblast marker genes. The findings show that PGE_2_ effects in fracture repair are complex and likely involve both stimulatory effects, through EP2 and EP4, and inhibitory effects mediated by EP1. Altogether, our findings establish antagonism of the EP1 receptor as a potentially important target to enhance fracture healing.

## Materials and Methods

### Experimental animals

All animal studies were done in accordance with and with approval of the University of Rochester Committee on Animal Resources. Wild-type (WT) C57BL/6J mice were purchased from Jackson Laboratories (Bar Harbor, ME, USA). The *EP1*^−/−^ mice (C57BL/6J background) were described previously.(31)

### Femoral fracture model

Closed femur fractures were created in 10-week-old *EP1*^−/−^ mice and WT C57BL/6J mice. Mice were anesthetized with 60 mg/kg of ketamine and 4 mg/kg of xylazine intraperitoneally. A 23-gauge needle (BD Medical Systems, Franklin Lakes, NJ, USA) was inserted into the length of medullary canal of the femur from the distal end, and a mid-diaphyseal fracture was created via three-point bending with an Einhorn device, as described previously.(32) Healing of the femur fracture was monitored using radiographs, which were obtained at 0, 7, 14, and 21 days under anesthesia using a Faxitron Cabinet X-Ray System (Faxitron X-Ray Corporation, Lincolnshire, IL, USA).

### Histology and analysis

The fractured femurs were collected on days 5, 7, 10, 14, 21, 28, and 35 after fracture. Excess muscle and soft tissue was excised. Four specimens from each group were fixed in 10% neutral buffered formalin. The specimens were decalcified for 21 days in 14% EDTA (pH 7.2), embedded in paraffin, and sectioned at a thickness of 3 µm. Levels were cut at depths of 30 µm for histomorphometric analysis (three levels per animal). The sections were stained using alcian blue hematoxylin/orange G eosin (ABH/OGE) and cytochemically for tartrate-resistant acid phosphatase (TRAcP). Total callus area, total cartilage area, and total woven bone area were quantified using a standardized eyepiece grid, as described previously.(33) Immunohistochemistry also was performed on these sections using a previously described method.(34) The mouse anti-EP4 antibody (Cayman Chemical, Ann Arbor, MI, USA) was used at a dilution of 1:200.

### Bone marrow cell culture

Bone marrow cells were isolated from 10-week-old *EP1*^−/−^ and C57BL/6J mice. Cells were cultured in 2 mL of α modified essential medium (α-MEM) containing 10% fetal bovine serum (FBS) at 5 × 10^6^ cells/well in 6-well plates. After 7 days in culture, the medium was replaced with medium containing 10 mM β-glycerophosphate and 50 mg/mL of ascorbic acid. This medium was changed every 2 days thereafter. On days 10, 12, 14, 17, and 21 after plating, cells were fixed for alkaline phosphatase and alizarin red staining or harvested for mRNA isolation using the RNeasy Mini Kit (Qiagen, Valencia, CA, USA). Cell proliferation was examined at day 7 using the Cell Proliferation ELISA, BrdU (colorimetric) immunoassay kit (Roche, Nutley, NJ, USA) according to the manufacturer's instructions. Cell viability was examined at day 7 using the CellTiter-Blue cell viability assay (Promega, San Luis Obispo, CA, USA) according to the manufacturer's instructions. Intracellular cAMP activity in the bone marrow cells was examined using the cAMP-Glo assay (Promega) according to the manufacturer's instructions.

### cDNA synthesis and quantitative real-time PCR

Mice were euthanized at 3, 7, 10, 14, 21, and 28 days after fracture (*n* = 4). Fracture callus tissue samples were carefully dissected from soft tissue and homogenized using the TissueLyzer (Qiagen). Total RNA then was extracted from the homogenized samples using the RNeasy Fibrous Tissue Midi Kit (Qiagen) according to the manufacturer's instructions. One-microgram aliquots of RNA were reverse transcribed into cDNA using the iScript cDNA Synthesis Kit (Bio-Rad, Hercules, CA, USA). Real-time PCR was performed on a Rotor-Gene 6000 real-time DNA amplification system (Qiagen) using the PerfeCTa SYBR Green SuperMix (Quanta BioSciences, Inc., Gaithersburg, MD, USA) according to the manufacturer's instructions. Real-time PCR analyses were performed using murine-specific primers for *β-actin*, *Col1a1*, *Col2a1*, *Col10a1*, *osteocalcin*, *ALP*, *Runx2*, *osterix*, *RANKL*, and *OPG* (see Table 1 for specific sequences).

**Table 1:** List of Oligonucleotide Primer Sequences for Real-Time PCR

*β-Actin*	5′-AGA TGT GGA TCA GCA AGC AG-3'
	5′-GCG CAA GTT AGG TTT TGT CA-3'
*Col1a1*	5′-GCC AAG GCA ACA GTC GCT-3'
	5′-CTT GGT GGT TTT GTA TTC GAT GAC-3'
*Col2a1*	5′-ACT GGT AAG TGG GGC AAG AC-3'
	5′-CCA CAC CAA ATT CCT GTT CA-3'
*Col10a1*	5′-CTT TGT GTG CCT TTC AAT CG-3'
	5′-GTG AGG TAC AGC CTA CCA GTT TT-3'
*Osteocalcin*	5′-AGG GAG GAT CAA GTC CCG-3'
	5′-GAA CAG ACT CCG GCG CTA-3'
*ALP*	5′- TGA CCT TCT CTC CTC CAT CC-3'
	5′- CTT CCT GGG AGT CTC ATC CT-3'
*Runx2*	5′-GCC GGG AAT GAT GAG AAC TA-3'
	5′-GGA CCG TCC ACT GTC ACT TT-3'
*Osterix*	5′-GTC AAG AGT CTT AGC CAA ACT C-3'
	5′-AAA TGA TGT GAG GCC AGA TGG-3'
*RANKL*	5′-CAC CAT CAG CTG AAG ATA GT-3'
	5′-CCA AGA TCT CTA ACA TGA CG-3'
*OPG*	5′-AGT CCG TGA AGC AGG AGT G-3'
	5′-CCA TCT GGA CAT TTT TTG CAA A-3'

### Spleen cell culture

Spleen cells were isolated from 2-month-old *EP1*^−/−^ and C57BL/6J mice and plated at 50,000 cells/well in α-MEM containing 10% FBS, 10 ng/mL of macrophage colony-stimulating factor (M-CSF; R&D Systems, Minneapolis, MN, USA), and 50 ng/mL of RANKL (R&D Systems) for 5 days, as described previously.(35) The cells were fixed with formalin and stained for TRAcP activity. Multinucleated TRAcP^+^ cells were counted as osteoclasts.

### Western blot analysis

Bone marrow cells were washed with cold phosphate-buffered saline (PBS) three times and lysed on ice in Golden Lysis Buffer (GLB) for 30 minutes, as described previously.(16) The soluble and insoluble portions in the cell lysate were separated by centrifugation at 13,000*g* for 30 minutes. The insoluble portion was discarded, and the protein concentration of the soluble portion was examined using the Coomassie Plus Protein Assay kit (Pierce Biotechnology, Rockford, IL, USA). Fifteen-microgram aliquots of protein extract were separated by SDS-PAGE. After transfer to a polyvinylidene fluoride (PVDF) membrane (Invitrogen, Carlsbad, CA, USA), the blots were probed with the following antibodies: anti-EP2 receptor, anti-EP4 receptor, and anti-β-actin (Sigma, St Louis, MO, USA) at a dilution of 1:1000. Alkaline phophatase–conjugated goat anti-rabbit and goat anti-mouse antibodies (Pierce Biotechnology) were used as secondary antibodies. The immune complexes were detected using an 4-nitro blue tetrazolium (NBT)/5-Bromo-4-Chloro-Indolyl-Phosphatase (BCIP) substrate (Pierce Biotechnology).

### Statistics

Results are shown as the mean ± SEM. Statistical significance was identified by Student's *t* tests and two-way ANOVA followed by Dunnett's tests; *p* values of less than .05 were considered significant.

## Results

### Fractures in *EP1*^−/−^ mice undergo accelerated endochondral bone formation and healing

**Fig. 1:**
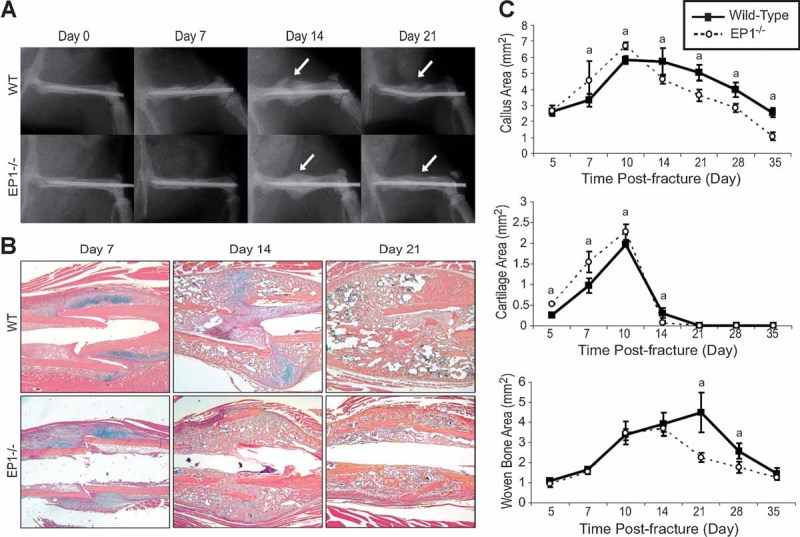
*EP1*^−/−^ fractures exhibit accelerated mineralization. Femur fractures were created in 10-week-old *EP1*^−/−^ mice and wild-type (WT) controls. Fractured femurs were harvested at 7, 14, and 21 days after fracture. Representative radiographs of fractured femurs demonstrate the increased mineralized callus in *EP1*^−/−^ mice on day 14 compared with the soft callus in wild-type fractures (*arrows*) (*A*). Accelerated remodeling on day 21 in *EP1*^−/−^ fractures is evident from the contracted callus versus the broad callus that remains in wild-type fractures (*arrows*). Representative histology (40× original magnification) of fractured femurs stained with alcian blue hematoxylin/orange G eosin show cartilage (*blue*) and bone (*orange*) formation (*B*). Histomorphometric measurements (total callus area, cartilage area, woven bone area) were made from 12 sections for each group (*n* = 4 mice/group) (*C*), and the results are shown as mean ± SEM. Statistical significance was assessed by two-way ANOVA followed by Dunnett's test; *a* indicates *p* < .05 compared with wild-type.

A murine femur fracture model was used to study fracture healing in the *EP1*^−/−^ mice. Fracture calluses from 10-week-old *EP1*^−/−^ mice and age- and sex-matched wild-type control mice were examined by radiographic analysis and histologic staining at days 7, 14, and 21 following fracture. Both radiographs and histology demonstrated accelerated fracture healing in *EP1*^−/−^ mice compared with wild-type mice (Fig. 1). On days 7 and 10 following fracture, the *EP1*^−/−^ mice had increased callus area and more cartilage formation than wild-type mice (Fig. 1*B, C*). By day 14, fractures in the *EP1*^−/−^ mice were healed, and cartilage tissue was essentially absent in the callus. In contrast, healing was incomplete in 14-day-old fractures in wild-type mice, where a central area of cartilage tissue persisted (Fig. 1*A*–*C*). In addition to the accelerated mineralization, fractures in the *EP1*^−/−^ mice underwent more rapid remodeling. Callus area peaked on day 10 in the *EP1*^−/−^ mice and then decreased progressively. In contrast, peak callus area occurred on days 10 to 14 in wild-type mice. Beginning on day 14, wild-type mice had increased callus area, and this persisted throughout the 35-day time course.

The mineralized calluses were examined by micro–computed tomography (µCT) analysis (Supplemental Fig. 1). Consistent with the results from radiographic analysis and histologic staining, 14-day-old fractures in the *EP1*^−/−^ mice were completely bridged by a calcified callus, whereas fractures in wild-type mice still exhibited a large gap (Supplemental Fig. 1*A, B*). On day 21 after fracture, femurs in the *EP1*^−/−^ mice almost returned to normal shape compared with the large callus seen in wild-type mice. Reconstruction of the µCT data showed that fracture calluses from the *EP1*^−/−^ mice had higher bone mineral density (BMD) than those of wild-type mice after 14 days of healing (Supplemental Fig. 1*B*). Torsion testing was performed to examine the strength of the femurs from the *EP1*^−/−^ and wild-type control mice at day 21 after fracture (Supplemental Fig. 2). Femurs from the *EP1*^−/−^ mice had higher ultimate torque, torsional rigidity, ultimate rotation, and torsional energy to failure.

**Fig. 2:**
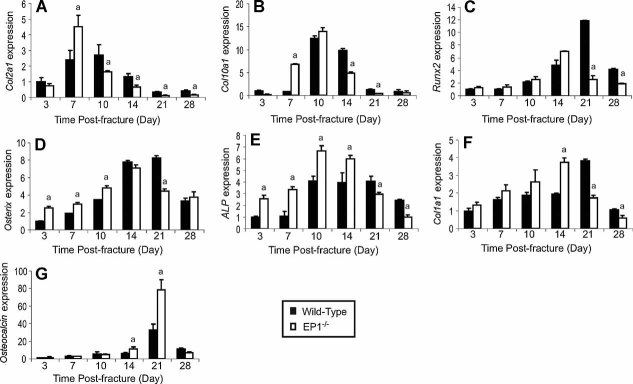
The expression of genes involved in chondrogenesis and osteogenesis is altered in *EP1*^−/−^ mice. Total RNA was extracted from *EP1*^−/−^ mice and wild-type controls (*n* = 4) at various time points after fracture. Real-time RT-PCR was performed as described under “Materials and Methods” and normalized to *β-actin* expression. The following primer sets were used: *Col2a1* (*A*), *Col10a1* (*B*), *Runx2* (*C*), *osterix* (*D*), *ALP* (*E*), *Col1a1* (*F*), and *osteocalcin* (*G*). Statistical comparisons at each time point were performed using two-way ANOVA followed by Dunnett's test; *a* indicates *p* < .05.

Quantitative real-time PCR analysis was performed to determine the expression of genes associated with endochondral bone formation in the fracture callus tissue from wild-type and *EP1*^−/−^ mice (Fig. 2*A, B*). Both *collagen, type II* (*Col2a1*) and *collagen, type X* (*Col10a1*) expression peaked earlier during fracture repair in the *EP1*^−/−^ mice than in the wild-type mice, so expression levels of these genes were significantly higher in the *EP1*^−/−^ mice 7 days after fracture (Fig. 2*A, B*). In contrast, peak expression of *Col2a1* and *Col10a1* occurred later in wild-type fractures, so they were increased significantly in the wild-type mice compared with the *EP1*^−/−^ mice at between 14 and 21 days after fracture. The more rapid appearance of these cartilage-specific genes and their earlier disappearance from the fracture callus are consistent with accelerated endochondral bone formation in the *EP1*^−/−^ mice.

### Osteoblast differentiation is accelerated in fractures in *EP1*^−/−^ mice

The expression of genes involved in osteoblast differentiation also was examined in the callus tissue of fractures from *EP1*^−/−^ and wild-type mice (Fig. 2*C–G*). Expression levels of the osteoblast-specific transcription factors *Runx2* and *osterix* were accelerated in fracture calluses from the *EP1*^−/−^ mice (Fig. 2*C, D*). Their expression peaked on day 14 in the *EP1*^−/−^ mice, whereas in wild-type callus tissue these genes had maximal expression on day 21 (Fig. 2*C, D*). Expression levels of the osteoblast differentiation markers *alkaline phosphatase* (*ALP*), *collagen, type I* (*Col1a1*), and *osteocalcin* also were elevated earlier in fractures from the *EP1*^−/−^ mice, consistent with accelerated osteoblast differentiation (Fig. 2*E–G*). Furthermore, the magnitude of both *ALP* and *osteocalcin* was higher in fractures in the *EP1*^−/−^ mice. Specifically, *ALP* expression in fractures from the *EP1*^−/−^ mice was significantly higher than in wild-type mice from day 3 to day 14, with peak expression occurring 10 days after fracture. In contrast, *ALP* expression in fractures in wild-type mice had a broad peak of maximal expression at between 10 and 21 days, and the expression levels in fractures from wild-type mice were increased compared with those from the *EP1*^−/−^ mice at 21 and 28 days. Expression levels of *Col1a1* and *osteocalcin* also were elevated earlier in fractures in the *EP1*^−/−^ mice compared with wild-type mice. These findings suggest that osteoblasts undergo a more rapid differentiation in *EP1*^−/−^ mice.

**Fig. 3:**
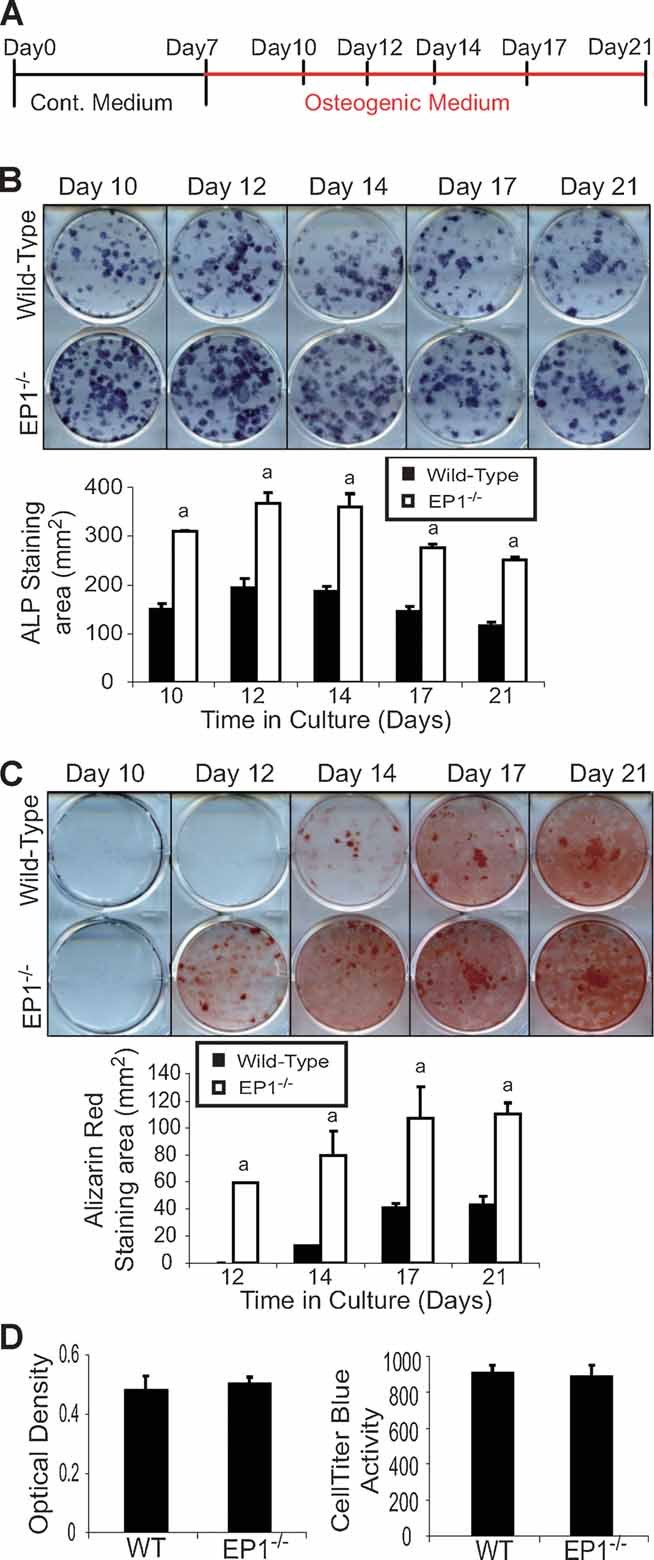
*EP1*^−/−^ bone marrow cultures have accelerated osteoblast differentiation. Bone marrow cells were isolated from 10-week-old *EP1*^−/−^ mice and control C57BL/6J mice. (*A*) Experimental design for in vitro osteoblast experiments. Cells were cultured in 2 mL of α-MEM containing 10% FBS at 5 × 10^6^ cells/well in 6-well plates. After 7 days, the medium was replaced with medium containing β-glycerophosphate and ascorbic acid to induce osteoblast differentiation. Cells were harvested on days 10, 12, 14, 17, and 21 after plating for alkaline phosphatase (*B*) and alizarin red staining (*C*). Cell proliferation rate was examined by BrdU incorporation and a CellTiter-Blue cell viability assay on day 7 (*D*). Statistical comparisons were performed using ANOVA. Significance was denoted by the symbol *a* (*p* < .05).

To determine whether mesenchymal stem cells from *EP1*^−/−^ mice have enhanced osteogenic potential, bone marrow progenitor cells extracted from *EP1*^−/−^ and wild-type mice were isolated and placed in culture. After 7 days, osteogenic medium containing β-glycerol-phosphate (BGP) and ascorbic acid was added to the cultures, and alkaline phosphatase and alizarin red staining was performed subsequently over time (Fig. 3*A*). Alkaline phosphatase staining showed that more colonies formed with increased alkaline phosphatase present in the *EP1*^−/−^ bone marrow stem cell cultures (Fig. 3*B*). Similarly, alizarin red staining was increased in the *EP1*^−/−^ bone marrow progenitor cell cultures compared with control cultures, consistent with accelerated bone nodule formation and mineralization (Fig. 3*C*). A BrdU incorporation assay, performed on day 7, showed that the *EP1*^−/−^ and wild-type bone marrow cells have similar rates of proliferation (Fig. 3*D*). Additionally, a CellTiter-Blue cell viability assay (Promega, San Luis Obispo, CA, USA) confirmed that a similar number of viable cells was present in both cultures on day 7 (Fig. 3*D*).

**Fig. 4:**
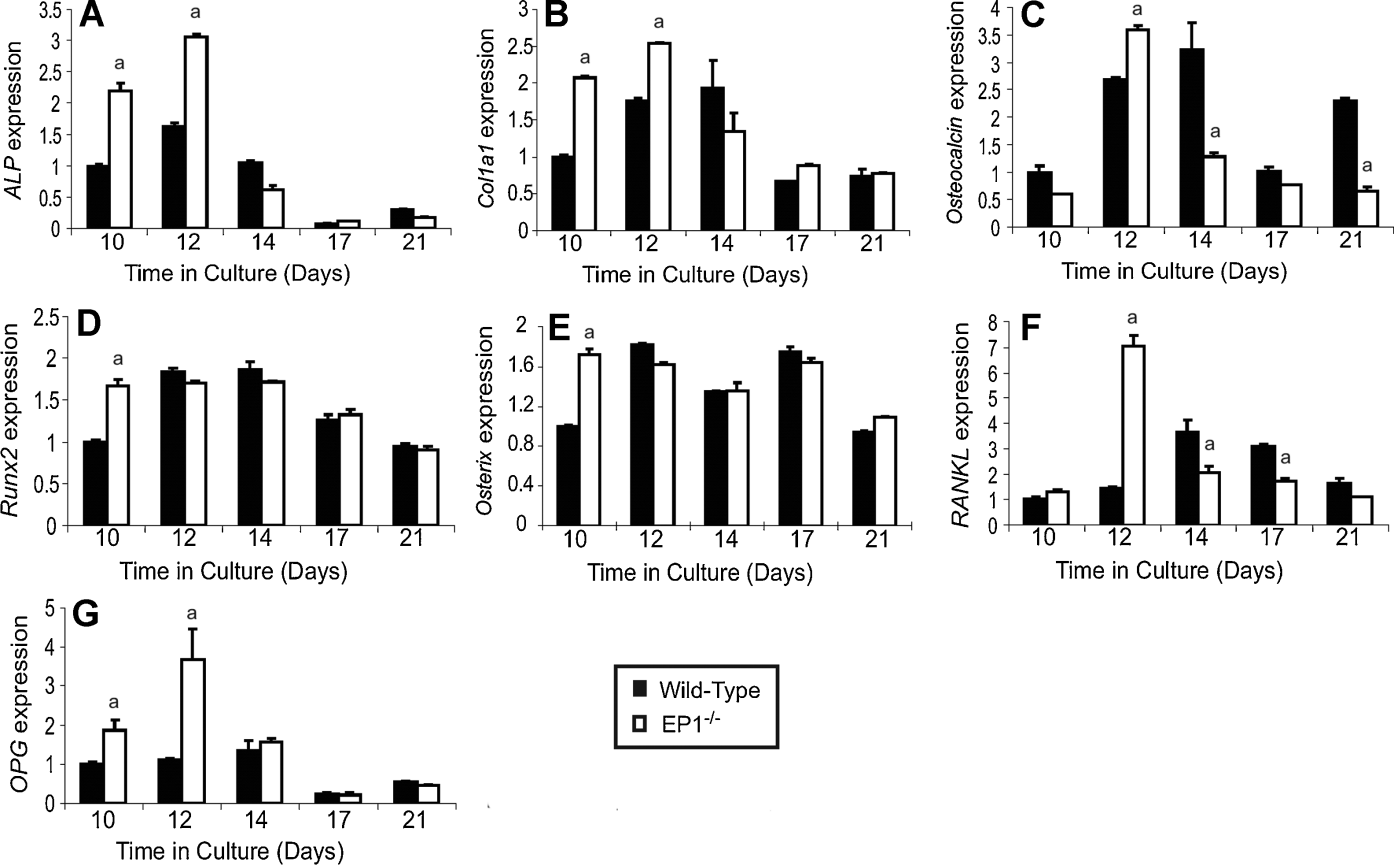
*EP1*^−/−^ bone marrow cells have enhanced expression of osteoblast genes in culture. Bone marrow cells were isolated and cultured as described for Fig. 3. Total RNA was harvested after 10, 12, 14, 17, and 21 days in culture, and RT-PCR was performed using the primers listed in Table 1. Statistical comparisons were performed using ANOVA at each time point. The symbol *a* indicates *p* < .05.

Total RNA was extracted from the bone marrow cultures to examine the expression of genes associated with osteoblast differentiation. *EP1*^−/−^ bone marrow stem cell cultures had enhanced early expression of *ALP*, *Col1a1*, *osteocalcin*, *Runx2*, and *osterix*, consistent with accelerated osteoblastogenesis, compared with bone marrow cells from wild-type mice (Fig. 4*A–E*). *EP1*^−/−^ bone marrow stem cell cultures also had increased expression of *RANK ligand* (*RANKL*) and *osteoprotegerin* (*OPG*) (Fig. 4*F, G*).

### Fractures in *EP1*^−/−^ mice have accelerated remodeling owing to enhanced osteoclast-inducing signals

**Fig. 5:**
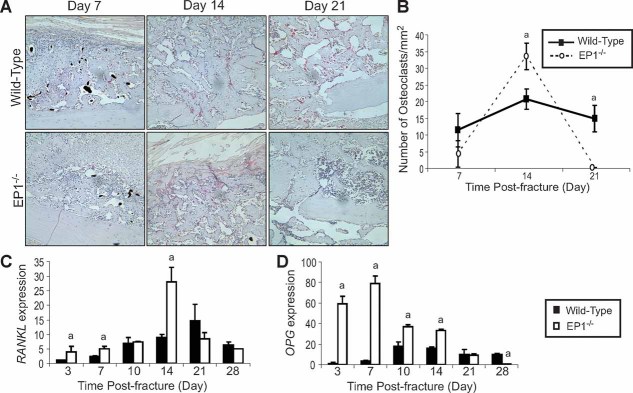
*EP1*^−/−^ fractures have accelerated bone remodeling. TRAcP staining was performed on sections of fractured femurs, and representative photomicrographs are shown at 200× original magnification (*A*). Osteoclast numbers were counted in 12 sections per group (*n* = 4 mice/group), and mean ± SEM is shown (*B*). *RANKL* (*C*) and *OPG* (*D*) RNA levels in the fracture callus were examined by RT-PCR. Statistical comparisons were performed using two-way ANOVA followed by Dunnett's test; *a* indicates *p* < .05.

Since histology and radiographic examination suggested that the fractures in the *EP1*^−/−^ mice underwent more rapid remodeling (Fig. 1), the fracture calluses were stained for the expression of TRAcP. The number of osteoclasts was increased by 58% (*p* < .05) in day 14 fracture calluses from *EP1*^−/−^ mice compared with wild-type mice, consistent with accelerated bone remodeling (Fig. 5*A*). By day 21, fractures in wild-type mice contained similar numbers of osteoclasts as observed in day 14 fractures in *EP1*^−/−^ mice. Surprisingly, few osteoclasts were observed in 21-day-old fractures from *EP1*^−/−^ mice (Fig. 5*A, B*). Total RNA was extracted from the calluses at various times, and RT-PCR was performed to measure the expression of the osteoclast-inducing gene *RANK ligand* (*RANKL*) and its soluble receptor antagonist *osteoprotegerin* (*OPG*). Fracture calluses from *EP1*^−/−^ mice had a higher overall level of expression of both *RANKL* and *OPG*, and both genes were elevated earlier in fractures in *EP1*^−/−^ mice compared with wild-type mice (Fig. 5*C, D*). The maximum expression levels of *RANKL* occurred at 14 days in fracture tissue from *EP1*^−/−^ mice and at 21 days in wild-type mice. While *RANKL* had a sharp peak of expression in facture tissue from *EP1*^−/−^ mice, the expression in wild-type mice had a lower magnitude and was more sustained, consistent with the observed differences in osteoclast numbers in the fracture calluses.

**Fig. 6:**
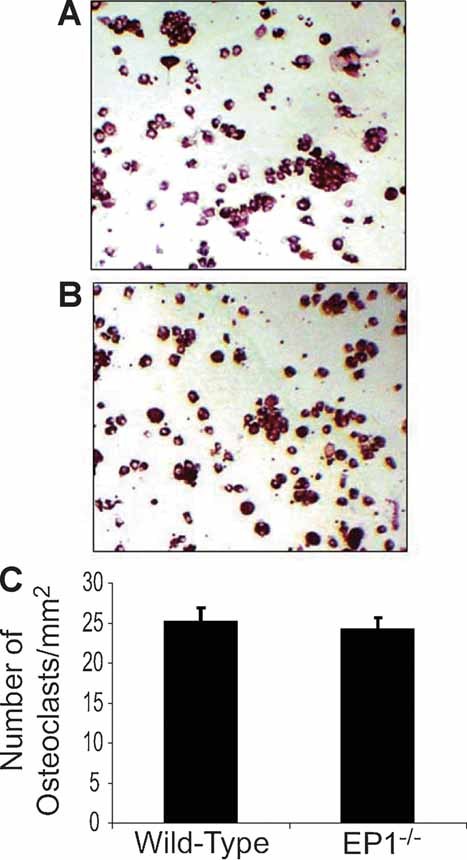
Increased osteoclastogenesis in *EP1*^−/−^ mice is not a cell-autonomous process. Splenocytes were isolated from 2-month-old *EP1*^−/−^ mice and control C57BL/6J mice. Cells were cultured at 50,000 cells/well in α-MEM containing 10% FBS and 10 ng/mL of M-CSF in 96-well plates. Then 50 ng/mL of RANKL was added to induce osteoclastogenesis. Five days after culture, TRAcP staining was performed (*A*, *B*). Representative photographs are shown at 100× original magnification. Similar numbers of osteoclasts were observed in *EP1*^−/−^ and wild-type cultures (*C*). The data in panel *C* represent the mean of four different experiments (8 culture wells per group per experiment).

To determine whether the accelerated osteoclast formation observed in *EP1*^−/−^ fractures is due to a cell-autonomous effect in osteoclast precursors from the *EP1*^−/−^ mice, spleen cells were isolated from *EP1*^−/−^ and wild-type mice and were placed in cell culture (Fig. 6). TRAcP staining performed on day 5 showed that similar numbers of osteoclasts formed in both *EP1*^−/−^ and wild-type cultures (Fig. 6). This result suggests that the accelerated osteoclast formation in *EP1*^−/−^ fractures is driven primarily by signals from enhanced osteoblastogenesis.

### *EP1*^−/−^ bone marrow mesenchymal stem cells do not have increased expression or activation of the EP2 or EP4 receptors

**Fig. 7:**
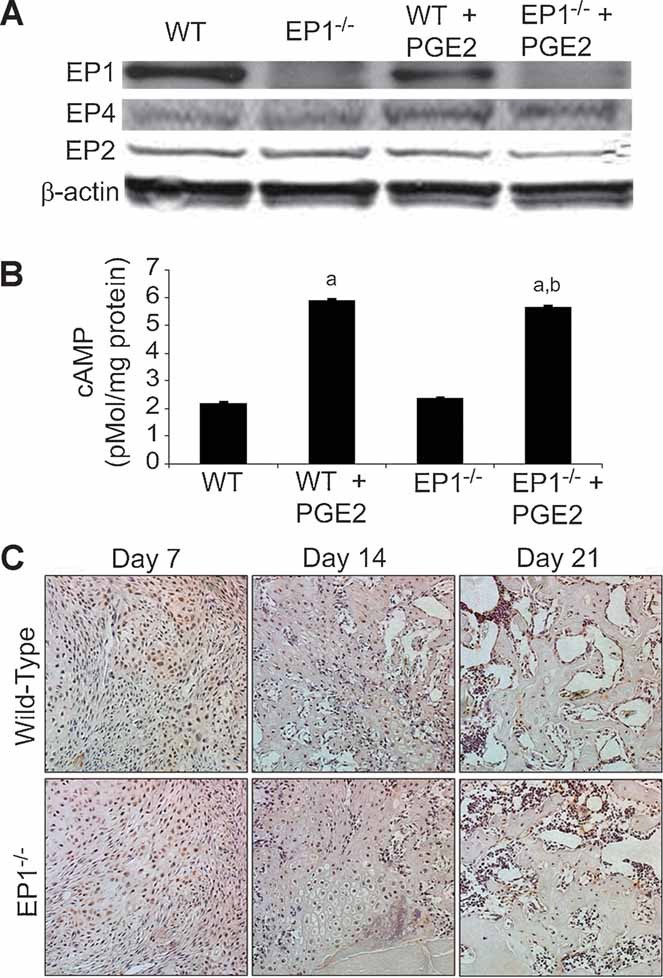
EP2 and EP4 signaling are not altered in *EP1*^−/−^ cells. Bone marrow cells were isolated from 10-week-old *EP1*^−/−^ mice and wild-type mice. After culturing for 7 days, cells were treated with PGE_2_ (3 µM) or vehicle for 24 hours. Total protein was extracted from the cultured cells, and Western blotting was performed using specific antibodies against the EP1, EP2, and EP4 receptors (*A*). A cAMP-Glo assay was performed on the bone marrow cells from 10-week-old *EP1*^−/−^ and wild-type mice (*B*). Immunohistochemistry was performed using specific antibodies against the EP4 receptors on the femur fracture tissue samples collected from 10-week-old *EP1*^−/−^ and wild-type mice at 7, 14, and 21 days after fracture, and representative photomicrographs are shown at 200× original magnification (*C*). Statistical comparisons were performed using Student's *t* test; *a* indicates *p* < .05 compared with wild-type, and *b*indicates *p* < .05 compared with *EP1*^−/−^. β-Actin served as the loading control.

Since prior work has established that activation of the EP2 or EP4 receptors can accelerate fracture healing, we performed experiments to confirm that deletion of EP1 does not result in the increased expression or activity of the EP2 and/or EP4 receptors. Protein levels of the EP2 and EP4 receptors in *EP1*^−/−^ and wild-type bone marrow cultures were examined. While *EP1*^−/−^ bone marrow cells had no expression of EP1, the expression of EP2 and EP4 was unchanged compared with bone marrow cells from wild-type mice (Fig. 7*A*). Additional experiments were performed to examine whether signaling through the EP2 and EP4 receptors was altered in the absence of EP1. Following administration of PGE_2_, cAMP levels were similarly enhanced in bone marrow cell cultures from wild-type and *EP1*^−/−^ mice (Fig. 7*B*). Finally, immunohistochemistry was used to examine the expression of the EP4 receptor in fractures from wild-type and *EP1*^−/−^ mice. Similar levels of expression were observed in the fractures from *EP1*^−/−^ and wild-type mice at 7, 14, and 21 days (Fig. 7*C*). Thus the accelerated fracture healing observed in the absence of EP1 was independent of the EP2 and EP4 receptors.

## Discussion

Despite the known importance of COX-2/PGE_2_ on fracture healing and bone repair, this study is the first to define the role of the EP1 receptor in this process. Fractures in *EP1*^−/−^ mice have accelerated chondrogenesis, chondrocyte maturation, endochondral bone formation, osteoblast differentiation, and bone remodeling. Gene expression studies from fracture callus tissues and bone marrow mesenchymal stem cell cultures demonstrated an enhanced rate of chondrocyte and osteoblast differentiation in *EP1*^−/−^ mice. The effect appears to be directly due to the EP1 receptor because neither differences in EP2 or EP4 receptor expression nor activity, as measured by intracellular cAMP levels, were observed between wild-type and *EP1*^−/−^ bone marrow cells. The findings show that the EP1 receptor is a negative regulator in bone repair. Since both the EP2 and EP4 receptors accelerate bone repair, the findings suggest that the overall effect of COX-2/PGE_2_ on the process of healing is determined by the relative activation of these various receptors.

Nonsteroidal anti-inflammatory drugs (NSAIDs), which are used widely as pain killers in fractures and other conditions, act by inhibiting COX-1 and COX-2. These drugs have been reported to impair the rate of fracture healing in humans.(2,4,17) Prior work by others and us has clearly established a role for COX-2/PGE_2_ in fracture healing in animal models.(7,10,11) PGE_2_ exerts anabolic and catabolic effects through its four receptor subtypes EP1 through EP4.(16) Activation of EP2 and EP4 stimulates the production of cAMP through Gs, whereas activation of EP3 results in a decrease in cAMP levels through Gi, Gq, or Gs depending on the EP3 isoform.(16,17) In contrast, the EP1 receptor is involved in regulating intracellular calcium levels. All four receptor subtypes are expressed in the fracture callus area (data not shown). Injection of EP2 or EP4 agonist immediately after fracture accelerates the healing process.(20,21) EP2 and EP4 induce bone formation through the PKA pathway in contrast to EP3, which has been shown to inhibit bone formation in vitro.(15–17,19–21,23) These experiments advance our understanding of the role of COX-2/PGE_2_ on fracture healing by showing that in addition to the EP2 and EP4 receptors, the EP1 receptor also plays a major role in fracture healing.

Fracture healing is a complex process in which bone injury results in the recruitment of mesenchymal stem cells to the site of injury, with subsequent proliferation and differentiation into bone-forming cells.(10) Our findings show that the EP1 receptor acts as a negative regulator of this process because cell differentiation and fracture healing are accelerated in mice deficient in the EP1 receptor. Our in vivo findings in the femur fracture model established that *EP1*^−/−^ mice have accelerated chondrogenesis, chondrocyte maturation, and endochondral bone formation. Furthermore, *EP1*^−/−^ mice have enhanced osteoblast differentiation in the fracture callus.

The in vivo findings were confirmed in cultures of bone marrow stem cells in which osteoblast differentiation and mineralization of *EP1*^*−/−*^ bone marrow cells both were accelerated compared with cells from wild-type mice. The mesenchymal stem cell experiments were performed in marrow-derived cells that are only one component of the stem cell population that contributes to fracture healing.(36) Since it is possible that other mesenchymal stem cell populations, including those from the periosteum, surrounding musculature, and vascular-derived stem cells could behave differently, the findings will need confirmation in other cell populations undergoing osteoblast differentiation. The finding that the expression levels of the transcription factors *Runx2* and *osterix* were increased in cells and fracture calluses from *EP1*^−/−^ mice suggests an important role for the EP1 receptor in cell fate determination because both these transcription factors regulate osteoblastogenesis from mesenchymal precursor cells.(37,38) As an inhibitor of osteoblast differentiation from mesenchymal stem cells, EP1 has an important role in the tissue response to bone injury.

An apparent paradox involving the COX-2/PGE_2_ signaling pathway is that while marked effects are observed on fracture repair, essentially no abnormalities are observed on skeletal development. While *COX-2*^−/−^ mice have a profound impairment in the differentiation of cartilage and bone from mesenchyme, limb development proceeds normally in *COX-2*^−/−^ mice.(10,30) These observations suggest that while reparative processes recapitulate many of the events observed during development, the repair process depends on the activation of unique signaling pathways. In a similar manner, postnatal growth and development also have been shown to depend on unique signaling pathways. An example is *Smad3*^−/−^ mice, which have normal skeletons at birth and grow normally until approximately 3 weeks of age, at which time profound abnormalities of endochondral bone formation develop.(39) Thus it is not surprising that the *EP1*^−/−^ mice develop normally. While recent work in our laboratory has established that *COX-2*^−/−^ mice have subtle differences in bone morphology and bone mass, to date, no differences in bone metabolism have been identified in the *EP1*^−/−^ mice used in this study.(31,40)

There are two different models of *EP1* gene deletion. The *EP1*^−/−^ mice initially described were produced by homologous recombination using a targeting vector that replaced a 671-base-pair sequence involving exon 2.(41) However, since the *EP1* receptor gene locus in mouse overlaps with the *PKN* protein kinase gene on the antiparallel strand, exon 2 deletion also resulted in disruption of *PKN* expression.(42) PKN is activated by rho GTPase and by fatty acids, including arachidonate, and has a C-terminal region that is highly homologous to protein kinase C.(43) Although the role of PKN in bone development has not been studied, it is possible that PKN regulates bone formation independent of EP1. In order to disrupt EP1 while sparing the PKN locus, Guan and colleagues deleted *EP1* by introducing a premature in-frame stop codon into exon 2 by nucleotide substitution.(31) Since the latter mice were used in this study, the findings depend completely on the EP1 receptor.

In addition to enhanced bone formation, our findings also suggest that fracture remodeling was accelerated in *EP1*^−/−^ mice compared with wild-type mice. We performed cell culture experiments to determine whether deficiency of EP1 was associated with the potential for accelerated osteoclast formation through a cell-autonomous process. In cell culture, spleen cells from *EP1*^−/−^and wild-type mice had similar rates of osteoclastogenesis following treatment with M-CSF and RANKL. In contrast, the *EP1*^−/−^ fracture calluses and cultures of *EP1*^−/−^ bone marrow stem cells had both accelerated and increased expression levels of *RANKL*. Thus the data support a primary role for enhanced osteoblast differentiation and RANKL expression in the enhanced osteoclastogenesis and remodeling observed in the fractures in *EP1*^−/−^ mice.

Consistent with these histologic and molecular observations, µCT analysis of fracture callus bone volume and mineral density (Supplemental Fig. 1) demonstrated an accelerated reduction in callus volume in *EP1*^−/−^ mice at 21 days, with a trend of increased mineral density, also suggesting accelerated remodeling of the fracture callus.

Biomechanical testing at 28 days following the fracture confirmed that the fractures in *EP1*^−/−^ mice healed more efficiently and with increased torsional strength and rigidity (Supplemental Fig. 1). The ultimate torque and torsional rigidity both were increased significantly in the *EP1*^−/−^ fractures. This confirms the fact that although *EP1*^−/−^ fractures have reduced callus area compared with wild-type mice late in the healing process, the fractures have enhanced biomechanical strength owing to increased bone remodeling. Since torsional strength in torsion is inversely related to the cross-sectional area of the callus,(44) the increased strength despite the reduced callus area and volume in the *EP1*^−/−^ fracture callus is indicative of higher-quality bone.

Compensatory induction of EP2 or EP4 signaling is a possible explanation for why *EP1*^−/−^ mice exhibit increased bone repair. However, no differences were found between wild-type and *EP1*^−/−^ bone marrow cells in either EP2 or EP4 receptor level or intracellular cAMP activity following stimulation with PGE_2_. This suggests that the phenotype of accelerated fracture healing in *EP1*^−/−^ mice is independent of changes in EP2 or EP4 signaling. The findings support a primary role for the EP1 receptor as a negative regulator of bone repair.

All together, these experiments show that the EP1 receptor is a negative regulator of fracture healing and demonstrate that the overall effect of COX-2/PGE_2_ signaling in fracture healing depends on the complex integration of signals from the various EP receptors. Our results suggest that inhibition of the EP1 receptor may increase fracture healing and support the importance of further studies to define the expression, regulation, and effects of this receptor in mesenchymal stem cells, chondrocytes, and osteoblasts during reparative processes.
