# Supplementary figures and images for: EP1−/− Mice Have Enhanced Osteoblast Differentiation and Accelerated Fracture Repair
Source: J Bone Miner Res. 2010 Oct 11;26(4):792–802. doi: 10.1002/jbmr.272 (PMC3179328; doi:10.1002/jbmr.272)

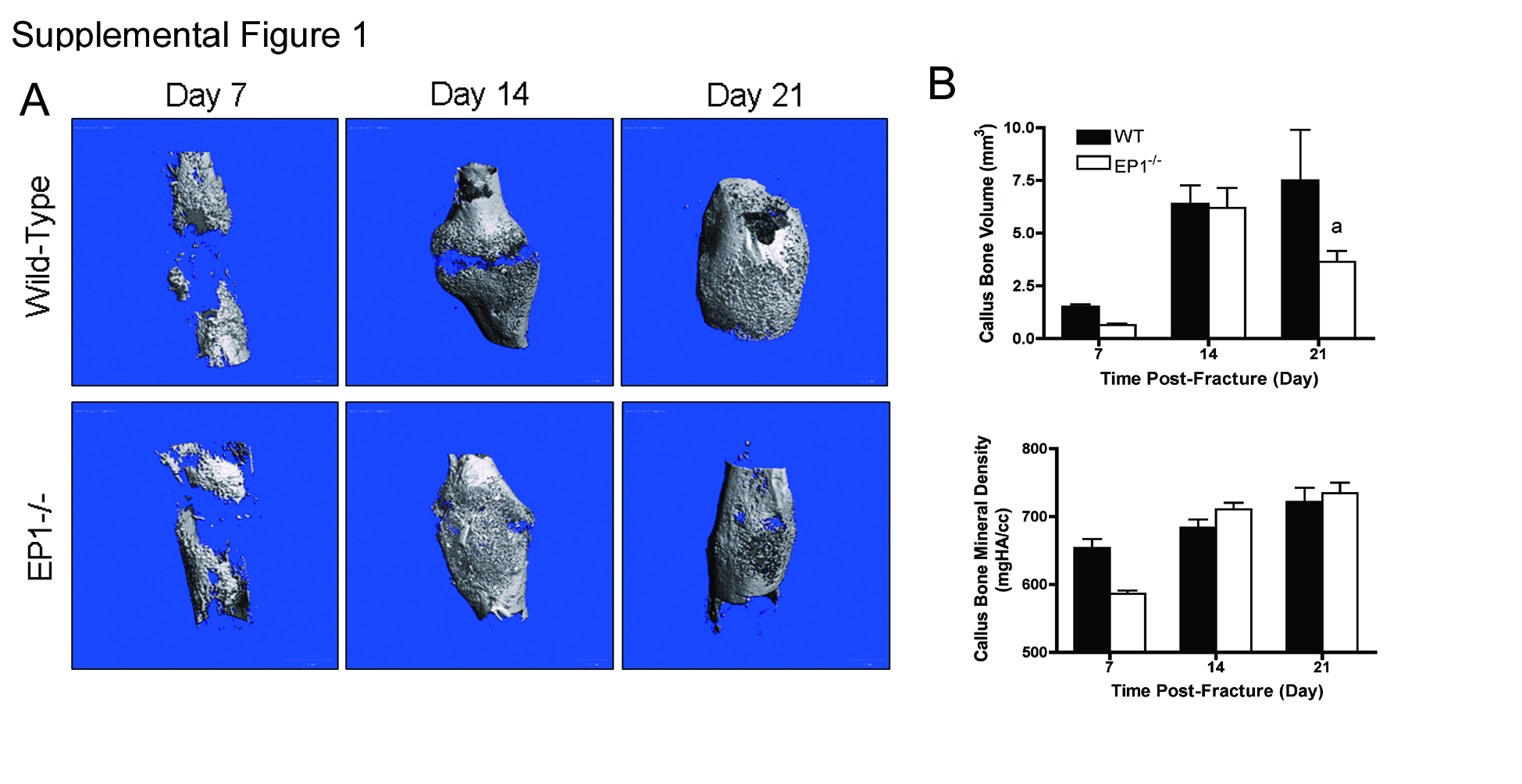

Supplement: Supplementary file 1 [file jbmr0026-0792-SD1.tif]
